# Clinical and economic consequences of switching from omalizumab to mepolizumab in uncontrolled severe eosinophilic asthma

**DOI:** 10.1038/s41598-021-84895-2

**Published:** 2021-03-09

**Authors:** Giovanna Elisiana Carpagnano, Emanuela Resta, Massimiliano Povero, Corrado Pelaia, Mariella D’Amato, Nunzio Crimi, Nicola Scichilone, Giulia Scioscia, Onofrio Resta, Cecilia Calabrese, Girolamo Pelaia, Maria Pia Foschino Barbaro

**Affiliations:** 1 Department of Basic Medical Sciences, Neuroscience and Sense Organs, Section of Respiratory Disease, University “Aldo Moro” of Bari, Bari, Italy; 2 Department of Economy, Translational Medicine and Health System Management, University of Foggia, Foggia, Italy; 3 AdRes - Health Economics and Outcomes Research, Turin, Italy; 4 Department of Health Sciences, Section of Respiratory Disease, University ‘‘Magna Græcia’’ of Catanzaro, Catanzaro, Italy; 5 Department of Clinical Medicine and Surgery, University “Federico II” of Naples, Section of Respiratory Disease, “Monaldi Hospital”, Naples, Italy; 6 Department of Internal Medicine and Specialist Medicine, Section of Respiratory Diseases, University of Catania, Catania, Italy; 7 Department of Health Promotion, Mother and Child Care, Internal Medicine and Medical Specialties, University of Palermo, Palermo, Italy; 8 Department of Medical and Surgical Sciences, Institute of Respiratory Diseases, University of Foggia, Foggia, Italy; 9 Department of Translational Medical Sciences, Section of Respiratory Disease, University of Campania “Luigi Vanvitelli”, Naples, Italy

**Keywords:** Respiratory tract diseases, Health care economics, Health care, Medical research

## Abstract

Severe asthma is burdened by frequent exacerbations and use of oral corticosteroids (OCS), which worsen patients’ health and increase healthcare spending. The aim of this study was to assess the clinical and economic impact of switching from omalizumab (OMA) to mepolizumab (MEP) in patients eligible for both biologics, but not optimally controlled by omalizumab. We retrospectively enrolled uncontrolled severe asthmatic patients who switched from OMA to MEP during the last two years. Information included blood eosinophil count, asthma control test (ACT), spirometry, serum IgE, fractional exhaled nitric oxide (FeNO), OCS intake, drugs, exacerbations/hospitalizations, visits and diagnostic exams. Within the perspective of Italian National Health System, a pre- and post-MEP 12-month standardized total cost per patient was calculated. 33 patients were enrolled: five males, mean age 57 years, disease onset 24 years. At OMA discontinuation, 88% were OCS-dependent with annual mean rate of 4.0 clinically significant exacerbations, 0.30 exacerbations needing emergency room visits or hospitalization; absenteeism due to disease was 10.4 days per patient. Switch to MEP improved all clinical outcomes, reducing total exacerbation rate (RR = 0.06, 95% CI 0.03–0.14), OCS-dependent patients (OR = 0.02, 95% CI 0.005–0.08), and number of lost working days (Δ = − 7.9, 95% CI − 11.2 to − 4.6). Pulmonary function improved, serum IgE, FeNO and eosinophils decreased. Mean annual costs were €12,239 for OMA and €12,639 for MEP (Δ = €400, 95% CI − 1588–2389); the increment due to drug therapy (+ €1,581) was almost offset by savings regarding all other cost items (− €1,181). Patients with severe eosinophilic asthma, not controlled by OMA, experienced comprehensive benefits by switching to MEP with only slight increases in economic costs.

## Introduction

Severe asthma in the era of personalized medicine can benefit from the newest therapeutic approaches that are changing the natural disease course. However, severe asthma still accounts for approximately half of asthma-associated healthcare costs^1^. The most important costs of severe asthma are mainly due to management of not controlled patients in terms of exacerbations, frequent access to health services, drug consumption, side effects of oral corticosteroids (OCS) use, treatment of comorbidities and losses from missed work and school days^2,3^. Most of these clinical aspects further extend beyond direct medical costs, and are also associated with personal problems leading to significant worsening of health-related quality of life (HRQoL), which negatively impacts on patients and caregivers^4^. Furthermore, an estimated one third of asthma-related deaths occur in patients previously hospitalized for exacerbations, thereby indicating a high mortality risk in severe uncontrolled asthma^3^.

GINA (Global Initiative for Asthma) guidelines recommend at step 5 the use of biologics before OCS, when maximum dosages of dual therapy based on inhaled corticosteroids (ICS) and long-acting β_2_-adrenergic agonists (LABA), eventually integrated by other controller drugs, do not allow to obtain an adequate disease control^2^. However, although more than half of severe asthmatic patients are eligible to at least one of the available biologics, most of these subjects are still not using such therapeutic agents, thus remaining uncontrolled. About both patient and physician concerns due to a poor knowledge about biologics, the cost of these new drugs discourages payer policies and often represents the main cause of their limited utilization in clinical practice^4^. However, real-life studies demonstrated that the pheno-endotype guided use of biologics is globally cost-effective in responders, thereby reducing overall costs of severe asthma management^3^.

For more than a decade, the only available biologic therapy for severe uncontrolled allergic asthmatics was omalizumab (OMA), sometimes prescribed also in patients who did not represent the ideal targets as their asthma was only partially driven by allergy^5^. OMA is a monoclonal antibody which prevents IgE from binding to its high-affinity receptor (FcεRI) found on mast cells and basophils, thus blocking upstream the inflammatory cascade of allergic asthma. Although OMA resulted to be effective in lowering clinical exacerbations and symptoms, thus improving lung function, sparing OCS and bettering quality of life (QoL)^6^, therapeutic effectiveness was only partial in some patients, so that the cost of this drug should be added to the burden of standard management of uncontrolled asthma^7^. The cost of an add-on biological treatment cannot be completely justified when it is not fully effective. This can happen when our therapeutic choices are not perfectly appropriate, with the consequence that a given biologic does not provide an adequate asthma control and further increases healthcare expenditures.

The introduction of new biologic therapies with different mechanisms of action and targets, such as mepolizumab (MEP), reslizumab (RES), benralizumab (BEN) and dupilumab (DUP), recently changed the therapeutic scenario of severe asthma making it possible for clinicians to operate a reasoned choice^6,8,9^. MEP, RES and BEN are three monoclonal antibodies that prevent the IL-5 binding to its receptor on eosinophils. This results in a downstream reduction of eosinophilic inflammation. In addition, through its afucosylated Fab fragment, BEN enhances the engagement of natural killer cells, resulting in antibody-directed cell-mediated cytotoxicity and eosinophil apoptosis. Finally, DUP is a monoclonal antibody that targets the IL-4α receptor and blocks signaling of both IL-4 and IL-13. Therefore this drug can act both upstream and downstream of the type 2 inflammatory cascade, thus inhibiting the production of IgE and the subsequent recruitment of inflammatory cells (effects mediated by IL-4) in addition to goblet cell hyperplasia and airway remodeling (effects mediated by IL-13). It is today imperative to select the biologic that could provide the best possible response by targeting the recognized biological pathway underlying the specific patient phenotype^10^. If, for example, OMA might be more effective in subjects with polyallergy, MEP, RES and BEN could be more effective in patients with elevated markers of eosinophic inflammation (i.e. elevated blood eosinophil counts and FeNO). DUP, on the other hand, could be more effective in more symptomatic patients or patients with polycomorbidity (i.e. atopic dermatitis and polyposis). Availability of several biologics also allows an eventual switch from one drug to another in unresponsive patients, with probable clinical and economic advantages. Several studies analyzed the cost-effectiveness of OMA and MEP, but contrasting results about this relevant topic have been obtained^3,5^. Although experience on switches from a biologic to another, motivated by poor efficacy of the first prescribed drug, are now common and several authors have described the clinical benefits provided by changing OMA with MEP in selected severe asthmatic patients^11,12^, to our knowledge no studies have been published about the relative economic impact.

The aim of the present study was therefore to explore, for the first time, the economic consequences detectable in subjects with severe eosinophilic allergic asthma, eligible for both these biologics, undergoing a switch from OMA to MEPO because they were not optimally controlled by OMA. In order to pursue this objective, we analyzed important clinical aspects driving asthma-related expenses referring to a 12-month standardized pre- and post- MEP total cost, within the perspective of Italian National Health System (NHS).

## Methods

### Patients

All patients > 18 years old with uncontrolled severe eosinophilic asthma, who referred to 7 asthma university clinics in Italy and switched from OMA to MEP during the last two years, were retrospectively enrolled in the present study. The relevant clinical parameters were collected at three time points: before starting OMA (pre-OMA), before starting MEP (pre-MEP), and after about one year of MEP (post-MEP). Collected information included blood eosinophil count, asthma control test (ACT) score, pulmonary function, serum IgE levels, fractional exhaled nitric oxide (FeNO), OCS intake, consumption of controller and rescue drugs, number of exacerbations and hospitalizations, unscheduled visits and diagnostic exams, and number of lost working days because of the disease. All patients started OMA due to severe uncontrolled allergic asthma, and switched to MEP if considered to be non-responders because of one or more of the following reasons: (i) they could not discontinue, or even needed to escalate, the daily dose of OCS; (ii) they experienced > 2 exacerbations or > 1 hospitalization per year. Switch to MEP was performed according to 2019 GINA guidelines^13^. This study was carried out according to the principles of the Declaration of Helsinki.

All patients had been firstly selected for treatment with omalizumab, and were then shifted to mepolizumab according to clinical practice and on the basis of all the criteria for eligibility approved by the European Medicines Agency. As in the OSMO study and how it is now routine in clinical practice, we performed a direct switch between the two drugs without a wash-out period. No changes in other regularly delivered therapies occurred in the post MEP period. The retrospective analysis of patient records reported in the present study was approved by the Policlinico Hospital of University of Bari “Aldo Moro” institutional review board. The clinical protocol was approved by the directors of all enrolled centers (Department of Basic Medical Sciences, Neuroscience and Sense Organs, University “Aldo Moro” of Bari; Department of Health Sciences, University ‘‘Magna Græcia’’ of Catanzaro; Department of Clinical Medicine and Surgery, University “Federico II” of Naples; Department of Internal Medicine and Specialist Medicine, University of Catania; Department of Health Promotion, Mother and Child Care, Internal Medicine and Medical Specialties, University of Palermo; Department of Medical and Surgical Sciences, Institute of Respiratory Diseases, University of Foggia; Department of Translational Medical Sciences, University of Campania “Luigi Vanvitelli”) and the study was conducted according to International Conference on Harmonization Guidelines on Good Clinical Practice. Patients signed a written informed consent for the treatment of personal data, which were anonymized.

### Economic evaluation

The annualized total cost of resource consumption, exacerbation management, pharmacologic therapies, and productivity loss were investigated during treatment periods with either OMA or MEP, in order to estimate the economic impact due to switching to MEP.

Cost of MEP treatment was evaluated considering 1 subcutaneous 100 mg vial every 4 weeks^26^, while the appropriate doses and administration frequencies of OMA were determined according to body weight and baseline serum IgE levels, measured before starting treatment^14^. The cost of both therapies for each treatment cycle was calculated according to the ex-factory price^15^, applying the mandatory discount (5% + 5% reduction) and price reductions negotiated among the pharmaceutical company, Local Health Units, and Hospital Units^16^. The costs of ICS/LABA, OCS, long-acting muscarinic antagonist (LAMA), short-acting β_2_-adrenergic agonists (SABA), and leukotriene inhibitors were calculated using the reference prices established by negotiation for each product package^17^, as well as for the specific posology referring to each patient (Supplementary Table S1). The cost of OCS therapy was calculated considering only prednisone, as the use of OCS was expressed in prednisone dose equivalents, assuming the best package fitting the daily dosage.

The cost of exacerbations treated only with drug therapy was calculated considering, if specified, the expense for one cycle of antibiotic therapy; no OCS extra therapy was considered since all patients were already treated with OCS. The tariff of € 280 for an emergency room visit was applied for severe exacerbations not needing hospitalization^18^, while for hospitalized exacerbations we considered the specific tariff of Diagnosis Related Group (DRG) reported in patient medical record^19^, detailed in hospital admission longer than 1 day (H) and day-hospital (DH). Specifically, DRG 96 “Bronchitis and asthma, age > 17 years with complications” (€ 2537 for H and € 198 for DH), DRG 97 “Bronchitis and asthma, age > 17 years without complications” (€ 1832 for H and € 197 for DH), and DRG 98 “Bronchitis and asthma, age < 18 years” (€ 1538 for H and € 185 for DH) were considered, respectively. Costs for specialist visits (€ 12.91, cod 89.01) and diagnostic exams were evaluated using National tariffs^19^. Tests and exams included spirometry (€ 37.18, cod 89.37.2), post bronchodilator test (€ 37.18, cod 89.37.4), serum IgE level measurement (€ 71.18, cod 90.68.1), FeNO detection (assumed same cost of spirometry), blood eosinophil number (€ 2.23, cod 90.62.5), and sputum eosinophil count (€ 27.17, cod 91.39.2). Each loss of a working day due to the disease was valued using the cost of paid and unpaid (household activities, caring for family members and others, and volunteering) work, specific for age and sex^20^, updated to 2019^21^ and reported in Supplementary Table S2. For non-workers (students, retired or unemployed workers), only the total length of hospital stay was multiplied by the cost of unpaid work.

### Statistical analysis

All resources (drug consumption, number of exacerbations, hospitalizations, unscheduled visits, diagnostic exams, and lost working days) were standardized to 12 months, divided the number of events by the person-years (PY) of follow-up pre-OMA, pre-MEP, and post-MEP.

Categorical variables are expressed as counts and percentages, continuous variables are summarized using mean and standard deviation (SD), and events (exacerbations with or without hospitalization) are expressed as absolute numbers and annual rates. Differences between pre- and post-MEP treatments were tested using generalized estimating equation (GEE) models^22^, with a covariate of treatment period; for dichotomous outcomes and count data, we assumed Binomial and Poisson distributions, respectively. Specific relative effect measures were also presented with 95% confidence intervals (CI): odds ratio (OR) for categorical outcomes, rate ratio (RR) for rates, and absolute difference for continuous outcomes, respectively. All statistical analyses were performed using STATA (StataCorp. 2017, release 15).

### Ethics approval and consent to participate

Ethical Committee approval was not needed because the drugs were prescribed according to current medical practice as part of the standard of care. The clinical protocol was approved by the directors of all enrolled centers (Department of Basic Medical Sciences, Neuroscience and Sense Organs, University “Aldo Moro” of Bari; Department of Health Sciences, University ‘‘Magna Græcia’’ of Catanzaro; Department of Clinical Medicine and Surgery, University “Federico II” of Naples; Department of Internal Medicine and Specialist Medicine, University of Catania; Department of Health Promotion, Mother and Child Care, Internal Medicine and Medical Specialties, University of Palermo; Department of Medical and Surgical Sciences, Institute of Respiratory Diseases, University of Foggia; Department of Translational Medical Sciences, University of Campania “Luigi Vanvitelli”) and the study was conducted according to International Conference on Harmonization Guidelines on Good Clinical Practice. Patients signed a written informed consent for the treatment of personal data, which were anonymized.

## Results

A total of 33 patients, previously treated with OMA and switched to MEP, were enrolled in the study. Mean age was 56.8 years (SD = 11.3), and the majority of participants were women (84.8%) with a family history of asthma (78.8%); mean duration of asthma was 23.7 years (SD = 12.2), all patients were sensitized to perennial allergens, and 69.7% were sensitized to other allergens. All baseline characteristics are detailed in Table 1.

**Table 1:** Summary of demographic and baseline clinical characteristics of the enrolled cohort (continuous data are presented as mean ± standard deviation, categorical data as absolute number and percentage in brackets).

Characteristics	N = 33
Age (years)	56.8 ± 11.3
Gender (female)	28 (84.8%)
Weight (kg)	69.5 ± 12.9
Body mass index (kg/m^2^)	27.6 ± 4.5
Former smoker	14 (42.4%)
Family history of asthma	26 (78.8%)
Duration of asthma (years)	23.7 ± 12.2
Atopy	33 (100%)
Sensitized to perennial allergens	33 (100%)
Sensitized to other allergens	23 (69.7%)
**Allergy comorbidities**	
Gastroesophageal reflux disease	18 (54.5%)
Sensitized to acetylsalicylic acid	6 (18.2%)
Nasal polyps	20 (60.6%)
Allergic rhinitis	13 (39.4%)
Bronchiectasis	6 (18.2%)
Obstructive sleep apnea syndrome	3 (9.1%)

**Figure 1:**
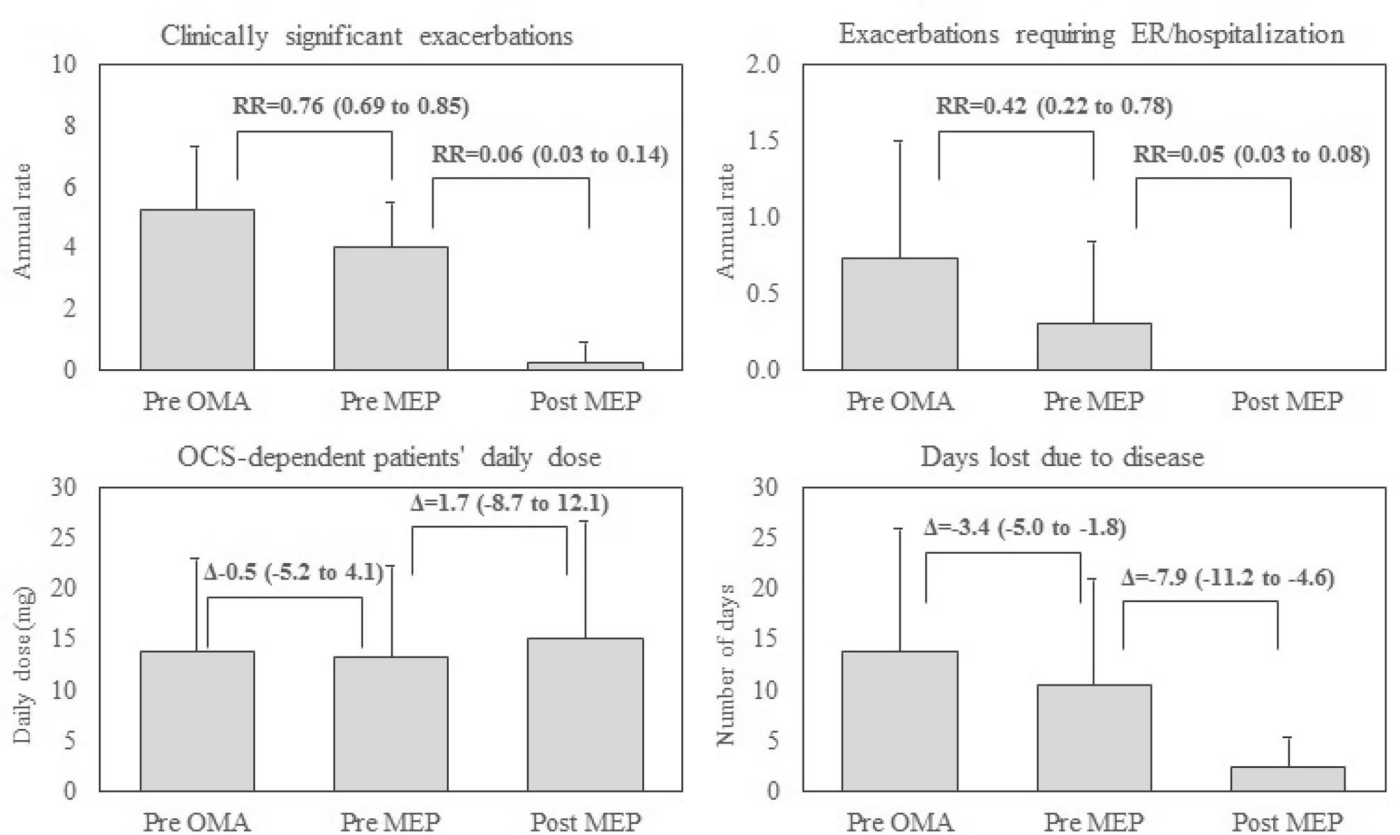
Pre-OMA, pre-MEP and post-MEP clinical evolutions in clinically significant exacerbation (top-left), exacerbations requiring ER or hospitalization (top-right), daily dose of OCS in OCS-dependent patients (bottom-left), and number of days lost due to the disease (bottom-right). Bars represent mean values, error bars represent standard deviations, and relative measures were expressed as mean (95% confidence interval). *Δ* mean difference, *ER* emergency room, *MEP* mepolizumab, *OMA* omalizumab, *OCS* oral corticosteroids, *RR* rate ratio.

There was no apparent difference between pre-OMA and pre-MEP periods with regard to almost all clinical parameters (Table 2 and Fig. 1), with the exception of slightly lower numbers referring to annual exacerbation rate (RR = 0.76, 95% CI 0.69 to 0.85), hospitalizations (RR = 0.42, 95% CI 0.22 to 0.78) and total number of lost working days due to disease (Δ = − 3.4, 95% CI − 5.0 to − 1.8).

**Table 2:** Clinical evolution of disease in the three periods considered in the analysis.

Parameter	Pre OMA	Pre MEP	Post MEP	Pre MEP vs pre OMA	Post MEP vs pre MEP
Patients OCS-dependent	27 (81.8%)	29 (87.9%)	4 (12.1%)	OR = 1.61 (0.84 to 3.11)	OR = 0.02 (0.005 to 0.08)
Patients with ACT ≥ 20	0 (0%)	0 (0%)	30 (90.9%)	–	OR = 2.43 (0.12 to 48.88)
ACT score	11.8 ± 3.35	13.9 ± 2.52	22.8 ± 3.28	Δ = 2.12 (1.16 to 3.08)	Δ = 8.91 (7.52 to 10.29)
FEV1 (L)	1.81 ± 0.65	1.77 ± 0.63	1.96 ± 0.67	Δ = − 0.02 (− 0.08 to 0.05)	Δ = 0.16 (0.09 to 0.24)
FEV1 (%)	75.1 ± 15.6	75.5 ± 17.8	82.3 ± 14.3	Δ = 0.01 (− 0.02 to 0.05)	Δ = 0.06 (0.03 to 0.08)
FVC (L)	2.63 ± 0.83	2.60 ± 0.83	2.75 ± 0.89	Δ = − 0.02 (− 0.1 to 0.07)	Δ = 0.13 (0.04 to 0.22)
FVC (%)	90.8 ± 14.3	88.1 ± 17.4	95.2 ± 14.5	Δ = − 0.02 (− 0.06 to 0.01)	Δ = 0.06 (0.00 to 0.12)
FEV1/FVC (%)	68.5 ± 11.8	67.9 ± 10.7	71.7 ± 10.5	Δ = 0.00 (− 0.03 to 0.02)	Δ = 0.03 (0.01 to 0.06)
IgE (UI/ml)	334 ± 249	354 ± 261	285 ± 229	Δ = 14.5 (− 27.4 to 56.4)	Δ = − 54.9 (− 95.2 to − 14.6)
FeNO (ppb)	36.7 ± 21.3	44.7 ± 24.1	32.8 ± 17.6	Δ = 6.8 (2.4 to 11.3)	Δ = − 11.3 (− 19.6 to − 3.0)
Eosinophils serum (cell/mcl)	499 ± 203	538 ± 223	71.6 ± 87.4	Δ = 31.6 (− 25.9 to 89)	Δ = − 466 (− 542 to − 391)

Continuous data are reported as mean ± standard deviation. Dichotomous data as absolute number (percentages), and relative measure as mean (95% confidence interval).

*ACT* asthma control test, *Δ* mean difference, *FeNO* exhaled nitric oxide, *MEP* mepolizumab, *OCS* oral corticosteroids, *OMA* omalizumab, *OR* odds ratio, *RR* rate ratio.

After almost 1 year of MEP therapy (mean 11.7 months, SD = 3.6), improvements in patient clinical conditions were highly evident in comparison to the period of treatment with OMA in regard to exacerbation rate (RR = 0.06, 95% CI 0.03 to 0.14), chronic OCS use (OR = 0.02, 95% CI 0.002 to 0.08), and lost working days due to disease (Δ = − 7.9, 95% CI − 11.2 to − 4.6), respectively. No subject needed hospitalization (Table 2 and Fig. 1). Daily OCS dose in OCS-dependent patients remained constant during the study period (Fig. 1). Furthermore, all main respiratory parameters improved (Table 2). Indeed, serum IgE levels decreased from 354 to 285 UI/mL (Δ = − 54.9, 95% CI − 95.2 to − 14.6), FeNO from 44.7 to 32.8 ppb (Δ = − 11.3, 95% CI − 19.6 to − 3.0), and blood eosinophil count from 538 to 71.6 cells/μL (Δ = − 466, 95% CI − 542 to − 391), respectively.

**Figure 2:**
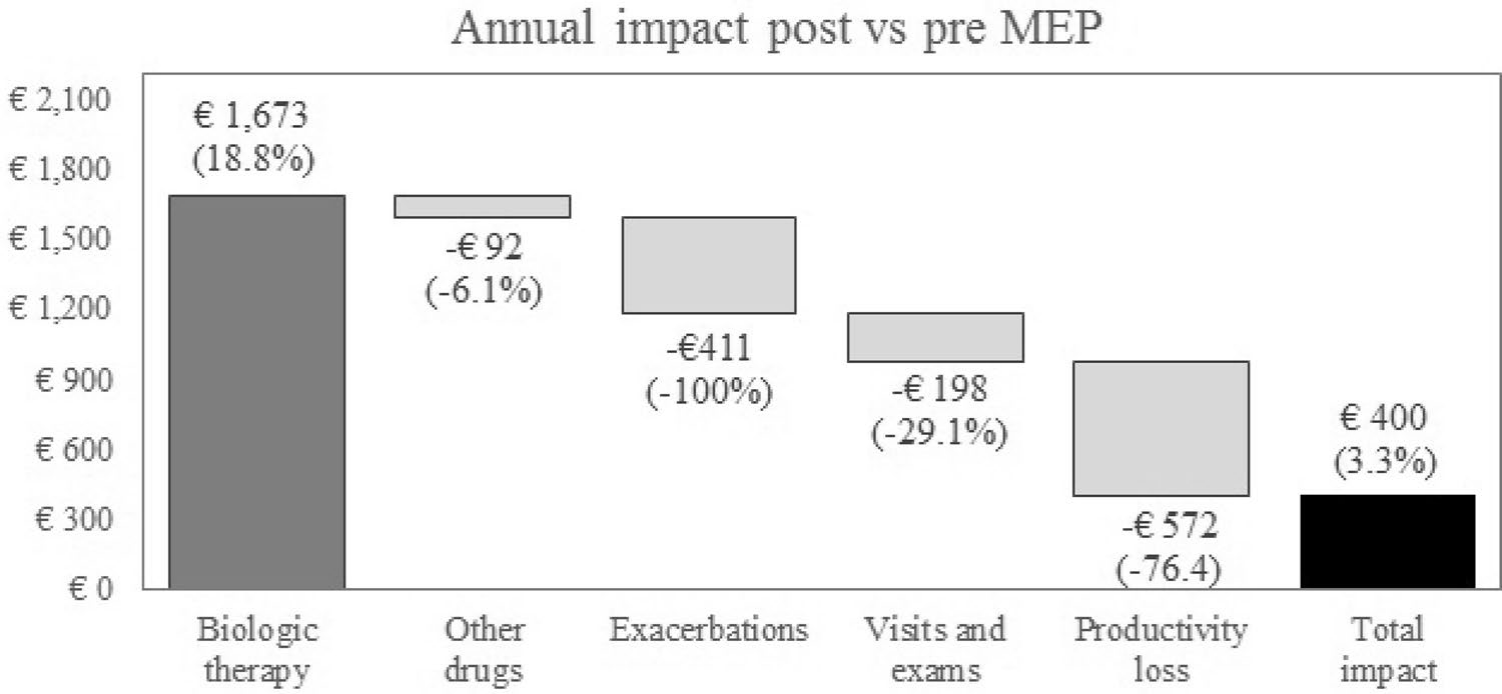
Mean annual economic impact of switching from OMA to MEP. *MEP* mepolizumab, *OMA* omalizumab.

The 12-month total costs referring to the two biological therapies were comparable (Table 3 and Fig. 2). The cost increment for drug therapy (€ 1581, 95% CI − 324 to 3,486) was due only to the cost of OMA (+ 18.8%), while the cost of the other drugs slightly decreased (− 6.1%). The impact of switching to a newer biologic treatment was almost completely offset by 100% saving in exacerbation management (€− 411, 95% CI − 727 to 94), 29.1% savings in visits/exams cost (€− 198, 95% CI − 289 to − 107), and 76.4% saving in productivity loss due to work absenteeism (€− 572, 95% CI − 858 to − 286).

**Table 3:** Total pre- and post-MEP annual costs.

	Pre MEP (€)	Post MEP (€)	Post vs pre MEP (€)
Total cost	12,239 ± 961	12,639 ± 88	400 (− 1,588 to 2,389)
Drug therapy	10,398 ± 912	11,979 ± 74	1,581 (− 324 to 3,486)
Exacerbations	411 ± 155	0	− 411 (− 727 to − 94)
Visits and exams	681 ± 32	483 ± 20	− 198 (− 289 to − 107)
Productivity loss	749 ± 173	177 ± 47	− 572 (− 858 to − 286)

Annual costs are reported as mean ± standard deviation, and annual delta as mean (95% confidence interval).

*MEP* mepolizumab, *OMA* omalizumab.

## Discussion

OMA has been for many years the only therapeutic choice for non-controlled severe allergic asthmatic patients, sensitized to perennial allergens. Nowadays, it is possible to switch OMA non-responding patients to other available biological treatments such as MEP, introduced in Italy since 2017^23^. In our study we enrolled 33 patients with severe eosinophilic allergic asthma, non-controlled by OMA, who switched to MEP. After 1 year of MEP therapy, we observed evident clinical benefits including significant decreases in asthma exacerbations, hospitalizations, OCS intake and working absenteeism. Reducing the risk of asthma exacerbations may be considered the most important outcome in obtaining ideal asthma control. Indeed, asthma exacerbations have been shown to be, not only the period of greatest risk and cause of anxiety to patients with asthma, but also the greatest source of costs to the health care systems in terms of days of work lost and asthma management^24^.

The most likely cause of unsatisfactory therapeutic response to OMA was probably related to the initial, obligatory choice of omalizumab due to unavailability of alternative biologics. In fact, at the beginning of this study and for more than the previous decade, OMA was the only available biologic therapy indicated for severe uncontrolled allergic asthmatics, and this drug was prescribed also in patients who did not represent the ideal targets, due to the lack of other alternatives^5^. Instead of OMA, some of these patients could likely have better benefited from MEP as first-line biologic treatment. For example, Massanari et al.^25^ have previously shown that OMA therapy results in blood eosinophil levels reduction. A possible explanation for the lacking in the reduction of eosinophils during OMA therapy in our patients could lie in the fact that actually the dominant pathway of their asthma endotype was that of IL-5. Furthermore, the high FeNO levels (> 25 ppb) showed by our patients at baseline, despite high doses of ICS, may be a further evidence of severe asthma IL-5-driven. Although the average BMI of our patients tends towards overweight, obesity was not a reason of non-response to OMA. Indeed, if we consider the interval between the minimum and maximum weight in kg of our patients (46–93), we can see that this is fully within the limits set by the tables for the dosage of this biologic.

The present study should not be considered as a comparison between an anti-IgE and an anti-IL-5 pharmacologic approach. Rather, in consideration of the increasing availability of biologic therapies, our aim is to stress the importance of gaining scientific evidence to support the rationale underlying the most effective drug choice for each specific patient.

The efficacy of MEP in patients not optimally controlled by OMA was previously evaluated in the OSMO trial^12^, and also in an Italian observational study^11^. Our results confirm the findings reported by both these studies. Moreover, we found significant decreases in exacerbation rate, hospitalization number, OCS-intake, and blood eosinophil count, which were very similar to those observed by Bagnasco^11^. The improvement in pre-bronchodilator FEV1 in our study was actually very slight and below the minimal clinically important difference (MCID) of 100 ml. We may have expected greater FEV1 increases, given the marked improvements detected with regard to other parameters. However, agreeing with the suggestion of the OSMO study^12^, we may suppose that patients with such severe and long-standing asthma might have undergone a remodeling process that limits their capacity to improve spirometry.

A few economic analyses regarding MEP have been published. A recent systematic literature review, aimed to investigate the cost-effectiveness of biological asthma treatments^3^, identified just one paper evaluating the long-term clinical and economic impact of adding MEP to standard treatments with ICS and other controller medications on severe eosinophilic asthmatic US patients^5^. Despite the significant improvement in quality of life, the estimated cost-effectiveness of MEP exceeded value thresholds. Similar results were detected by the authors of a more recent study^26^, but they conducted a further sensitivity analysis comparing MEP with OMA; in this “active” comparison, MEP resulted dominant (more effective and less costly) with respect to OMA. The annual cost of MEP, viewed from the US payer perspective, was also estimated on the basis of data published in MENSA trial^27^. The 12-month economic impact, not considering the cost of MEP, amounted to $ 1277 per patient, and hospitalization cost was the main expense. The difference with our findings was probably due to the different cost structure between US and Italy. Moreover, in the US study, indirect costs have not been included.

The potential economic benefit provided by MEP could be underestimated because of three study limitations emerging from our retrospective investigation. First, the sample size of our study was quite small (33 patients). It is therefore highly probable that, considering higher population samples, the savings in economic terms will be greater. Second, OCS use has important delayed clinical consequences^2,28^, such as infections, weight gain, diabetes and osteoporosis, and morbidity costs in OCS-dependent patients have a relevant impact on health systems^29,30^. Thus, a 12-month follow-up could not allow to really evaluate the potential savings due to OCS-intake reduction. Second, the number of lost working days because of severe asthma was reported only for employed subjects, whereas for unemployed patients we considered only the length of hospital stay (if a hospitalization occurred during the study). Therefore, working day losses were not recorded in non-hospitalized unemployed patients, even if productivity impairments were unavoidable in case of frequent exacerbations. Since female prevalence was greater than that observed in real-life^31^, gender distribution was highly unbalanced in our study, and it is well known that the employment rate is lower for women in comparison to men. Hence, it is very likely that a more balanced population could have allowed to verify greater savings with regard to the cost due to productivity loss. Furthermore, in recent years, for biologics have also become available easy-to-use subcutaneous auto-injectors or prefilled syringes that can be administered at home by the patients themselves or by their caregivers. Expanded patient access to at-home injection treatment possibilities with some biologics has the potential to further reduce costs of therapy limiting accesses to the GP's office and healthcare facilities for administration purposes.

To the best of our knowledge this is the first study investigating not only the clinical benefits, but also the economic impact of switching to MEP patients not optimally controlled by OMA. The economic analysis included both direct health care costs (drug consumption, hospitalizations, diagnostic exams, unscheduled visits) and indirect costs (absenteeism due to disease), that represented a significant portion of the total burden of asthma^32,33^. According to our findings, productivity loss accounted for about 22% of the annual cost due to uncontrolled asthma (excluding OMA cost); such percentage decreased to 9% after one year of treatment with MEP (excluding MEP cost).

In conclusion, our study provides evidence that patients with severe asthma, eligible for both OMA and MEP and not optimally controlled with the first of these two drugs, could improve their asthma control by switching to MEP. Once again we must remember that the purpose of our study was not a comparison between the two drugs' clinical efficacy, but to demonstrate the economic impact of a wrong choice, whether it was driven by the unavailability of other drugs or derived from an incorrect clinical assessment. The economic saving deriving from the therapeutic change is almost negligible for NHS, because the cost increment due to drug price can be partially compensated by savings arising from decreases in exacerbation frequency, hospitalization rate, OCS use, and work absenteeism.

## Supplementary Information

Supplementary Information

## Data Availability

The datasets used and/or analyzed during the current study are available from the corresponding author on reasonable request.
