# Supplementary material for: Clinical and economic consequences of switching from omalizumab to mepolizumab in uncontrolled severe eosinophilic asthma
Source: Sci Rep. 2021 Mar 9;11:5453. doi: 10.1038/s41598-021-84895-2 (PMC7943587; doi:10.1038/s41598-021-84895-2)
Supplement: Supplementary file 1 — Supplementary Information [file 41598_2021_84895_MOESM1_ESM.docx]

**Clinical and economic consequences of switching from omalizumab to mepolizumab in uncontrolled severe eosinophilic asthma.**

^1^Giovanna Elisiana Carpagnano, ^2^Emanuela Resta, ^3^Massimiliano Povero, ^4^Corrado Pelaia, ^5^Mariella D’Amato, ^6^Nunzio Crimi, ^7^Nicola Scichilone, ^8^Giulia Scioscia, ^1^Onofrio Resta, ^9^Cecilia Calabrese, ^4^Girolamo Pelaia , ^8^Maria Pia Foschino Barbaro

^1^Department of Basic Medical Sciences, Neuroscience and Sense Organs, Section of Respiratory Disease, University “Aldo Moro” of Bari, Bari, Italy

^2^Translational Medicine and Health System Management, Department of Economy, University of Foggia, Foggia, Italy^,^

^3^AdRes - Health Economics and Outcomes Research, Turin, Italy

^4^Department of Health Sciences, Section of Respiratory Disease, University ‘‘Magna Græcia’’ of Catanzaro, Catanzaro, Italy

^5^Department of Clinical Medicine and Surgery, University “Federico II” of Naples, Section of Respiratory Disease, “Monaldi Hospital”, Naples, Italy

^6^Department of Internal Medicine and Specialist Medicine, Section of Respiratory Diseases, University of Catania, Catania, Italy

^7^Department of Health Promotion, Mother and Child Care, Internal Medicine and Medical Specialties, University of Palermo, Palermo, Italy

^8^Department of Medical and Surgical Sciences, Institute of Respiratory Diseases, University of Foggia, Foggia, Italy

^9^Department of Translational Medical Sciences, Section of Respiratory Disease, University of Campania “Luigi Vanvitelli”, Naples, Italy

**Corresponding author**

Dr. Giulia Scioscia

Department of Medical and Surgical Sciences, Institute of Respiratory Diseases, University of Foggia, Foggia, Italy

[giulia.scioscia@unifg.it](mailto:giulia.scioscia@unifg.it)

**Supplementary Table S1.** Drug costs considered in the analysis (for SABA, leukotriene inhibitors and antibiotics the posology was assumed to be the same for all patients, while for ICS/LABA, OCS and LAMA is patient-specific).

| Category | Package | Price (€) |
| --- | --- | --- |
| ICS/LABA | BCL/F 100/6 mcg or 200/6 120 puff | 52.48 |
|  | BUF/F 160/4.5 mcg 120 puff | 46.84 |
|  | BUF/F 320/9 mcg 60 puff | 49.85 |
|  | BUF/F 160/4.5 mcg 120 puff or 320/9 mcg 60 puff (Turbohaler) | 62.41 |
|  | FP/F 250/10 mcg 120 puff | 73.98 |
|  | FP/S 250/50 mcg 120 puff (Diskus) | 49.00 |
|  | FP/S 500/50 mcg 60 puff (Spiromax) | 52.35 |
|  | FP/S 500/50 mcg 60 puff | 53.87 |
|  | FP/S 500/50 mcg 120 puff (Diskus) | 64.63 |
|  | FF/V 184/22 mcg 30 puff | 52.12 |
| OCS | prednisone 10 tablets, 5 mg | 1.26 |
|  | prednisone 20 tablets, 5 mg | 2.54 |
|  | prednisone 10 tablets, 25 mg | 4.58 |
| LAMA | tiotropium 18 mcg 30 doses | 42.39 |
|  | tiotropium 2.5 mcg 30 doses | 48.78 |
|  | aclidinium 322 mcg 60 doses | 48.26 |
|  | glicopirronium 44 mcg 30 doses | 45.37 |
| SABA | salbutamol100 mcg 200 doses ^†^ | 4.09 |
| Antileukotrienes | montelukast 28 tablets, 10 mg ^‡^ | 14.50 |
| Antibiotics | cefuroxima 500 mg 6 tablets ^§^ | 6.16 |

ICS: inhaled corticosteroids; LABA: long-acting beta-adrenoceptor agonist; LAMA: long-acting muscarinic antagonist; OCS: oral corticosteroids; BCL/F: beclometasone/formoterol; BUD/F: budesonide/formoterol; FP/F: fluticasone proprionate/formoterol; FP/S: fluticasone proprionate/salmeterol; FF/V: fluticasone furoate/vilanterol

^†^ Up to 800 mcg daily; ^‡^ 1 tablet daily; ^§^ 2 tablets daily for 7 days

**Supplementary Table S2.** Estimated age- and gender-specific yearly paid and unpaid production value.

PV: production value

| Annual PV | Gender | Age class | | | | | | | | |
| --- | --- | --- | --- | --- | --- | --- | --- | --- | --- | --- |
|  |  | 15-19 | 20-24 | 25-29 | 30-39 | 40-44 | 45-49 | 50-59 | 60-64 | 65+ |
| Paid (€) | male | 3,892 | 4,539 | 23,470 | 29,910 | 35,690 | 29,630 | 34,239 | 42,499 | 3,139 |
|  | female | 2,923 | 3,390 | 11,865 | 14,842 | 17,198 | 13,359 | 15,426 | 17,662 | 406 |
| Household (€) | male | 1,035 | | 2,439 | | | 4,213 | | | 5,987 |
|  | female | 3,326 | | 9,609 | | | 13,675 | | | 12,935 |
| Caring activities (€) | male | 222 | | 1,552 | | | 887 | | | 961 |
|  | female | 665 | | 3,770 | | | 1,478 | | | 887 |
| Volunteering activities (€) | male | 325 | | 108 | | | 217 | | | 325 |
|  | female | 108 | | 108 | | | 217 | | | 217 |
